# Gut Microbiota Co-microevolution with Selection for Host Humoral Immunity

**DOI:** 10.3389/fmicb.2017.01243

**Published:** 2017-07-04

**Authors:** Lingyu Yang, Shuyun Liu, Jinmei Ding, Ronghua Dai, Chuan He, Ke Xu, Christa F. Honaker, Yan Zhang, Paul Siegel, He Meng

**Affiliations:** ^1^Shanghai Key Laboratory of Veterinary Biotechnology, Department of Animal Science, School of Agriculture and Biology, Shanghai Jiao Tong UniversityShanghai, China; ^2^Department of Animal and Poultry Sciences, Virginia TechBlacksburg, VA, United States; ^3^Carilion ClinicRoanoke, VA, United States

**Keywords:** humoral immunity, gut microbiota, chicken, antibody titers, co-microevolution, host-microbe interactions

## Abstract

To explore coevolution between the gut microbiota and the humoral immune system of the host, we used chickens as the model organism. The host populations were two lines (HAS and LAS) developed from a common founder that had undergone 40 generations of divergent selection for antibody titers to sheep red blood cells (SRBC) and two relaxed sublines (HAR and LAR). Analysis revealed that microevolution of host humoral immunity contributed to the composition of gut microbiota at the taxa level. Relaxing selection enriched some microorganisms whose functions were opposite to host immunity. Particularly, Ruminococcaceae and *Oscillospira* enriched in high antibody relaxed (HAR) and contributed to reduction in antibody response, while *Lactobacillus* increased in low antibody relaxed (LAR) and elevated the antibody response. Microbial functional analysis showed that alterations were involved in pathways relating to the immune system and infectious diseases. Our findings demonstrated co-microevolution relationships of host-microbiota and that gut microorganisms influenced host immunity.

## Introduction

The gastrointestinal tract of an animal is colonized by a complex and abundant ensemble of microbes (Ley et al., 2008). Over millennia, microbes have evolved to coexist with their host and have a symbiotic role for avoiding elimination by the immune system (Garrett et al., 2010). In our previous studies, the heritability of microbiota was proposed to discern genetic from environmental factors that manage the gut microbiota and to potentially distinguish functionally important microbial members (Meng et al., 2014). In addition to heritabilities (Zhao et al., 2013; Meng et al., 2014; van Opstal and Bordenstein, 2015), we reported significant genetic correlations between specific microbes confirming that the genotype of the host was involved in microbe-microbe interactions (Zhao et al., 2013; Meng et al., 2014). The Christensenellaceae has been identified as the most highly heritable taxon and was significantly enriched in lean than obese humans (Goodrich et al., 2014). The heritability and significant genetic correlations of specific microbes suggested co-microevolution relationships for the host-gut microbiota axis. Therefore, increased attention should be focused on investigating co-microevolution mechanisms between gut microbiota and host genetic variation.

The gut microbiota is shaped by both the environment and genetics of the host, with the immune system in particular, considered to be involved in modulating the gut microbiota (Slack et al., 2009). The mutation of a single gene relating to immune response can have a considerable influence on the structure and composition of gut microbiota. For example, nucleotide-binding oligomerization domain protein 2 (NOD2), which recognizes peptidoglycan to enhance systemic innate immunity, can alter the composition of the gut microbiota (Clarke et al., 2010). The mutation of adaptor molecule MyD88 can impact gut microbial composition (Kubinak et al., 2015) and is associated with both a shift in bacterial diversity and a greater proportion of segmented filamentous bacteria in the small intestine (Larsson et al., 2012). Mice, deficient in activation-induced cytidine deaminase or recombination-activating gene 2 (Rag2) lack IgA (immunoglobulin A), exhibited predominant and persistent expansion of segmented filamentous bacteria (Suzuki et al., 2004). Thus, the immune system can have an important influence on the composition of the microbiota. Conversely, certain members of the microbiota can regulate the development of specific subsets of immune cell populations and establish the set-point for a steady-state immune system (Ivanov and Honda, 2012; Honda, 2015). Colonization of mice with segmented filamentous bacteria (SFB) induced an accumulation of T helper 17 cells and an increase in T helper 1 cells (Gaboriau-Routhiau et al., 2009). Clostridium has the ability for expansion of lamina propria and systemic T cells regulation (Atarashi et al., 2011). Polysaccharide A (PSA) of *Bacteroides fragilis* induces an IL-10 response in intestinal T cells, which prevents the expansion of T helper 17 cells and potential damage to the mucosal barrier (Round et al., 2011). Considering their long history of co-microevolution, while the gut microbiota and the host immune system bidirectional communicate in mutually beneficial ways, co-microevolution mechanisms between gut microbiota and the immune system of the host remains obscure. Especially how the antibody response involved in humoral immunity and gut microbiota modulate each other still remains unknown.

The factors that influence the mechanisms of the interactions between the host and the gut microbiota are likely to be small, and thus detecting them requires well controlled effects other than those of the host genotype. Thus, choosing a model organism that is maintained in a controlled environment should enhance our understanding of the relationships between gut microbiota and host genetic factors. The chicken, which bridges the evolutionary gap between mammals and reptiles, can serve as such an organism due to the characteristics of its less complex gut microbiota and minimal maternal effect.

To explore interactions between the host immune system and gut microbiota, we used next generation sequencing technology to investigate the composition of gut microbiota of White Leghorn chickens that had undergone long-term, bidirectional selection for a single immune trait (Siegel and Gross, 1980). In brief, 40 generation of selection from a common founder population, was performed for high (HAS) or low (LAS) antibody response 5 days post-injection of a non-pathogenic antigen, sheep red blood cells (SRBC) (Siegel et al., 1982; Boa-Amponsem et al., 1997). In addition, two sublines (HAR and LAR), in which selection was relaxed in generation 23 were also used in this study to investigate the response from gut microbiota after selection was relaxed. QTL mapping of host populations revealed 11 genomic regions associated with antibody response traits (Dorshorst et al., 2011). Furthermore, two SNP markers were significantly associated with day 5 antibody titers and overlap with *SEMA5A* and *TGFBR2*, respectively (Dorshorst et al., 2011; Lillie et al., 2017). Additionally, there were different haplotypes among lines in the major histocompatibility complex (MHC). The MHC haplotypes were fixed by generation 32 for B^21^ in HAS and B^13^ in LAS. The long-term divergent selection resulted in a 6.5-fold difference in antibody titers between HAS and LAS with modest regressions to the origin in the relaxed lines (Lillie et al., 2017).

## Materials and methods

### Animals and sample collections

Protocols used for this experiment were approved by the Institutional Animal Care and Use Committee at Virginia Tech. Lines for high antibody selection (HAS) and low antibody selection (LAS) were from a White Leghorn founder population that had been selected for high and low antibody response 5 days after a single intravenous injection of 0.1 mL of a 0.25% suspension of SRBC antigen administrated between 41 and 51 day of age (Siegel and Gross, 1980). Beginning in generation 23, HAS and LAS individuals were randomly chosen to produce sublines in which selection was relaxed. Those sublines, high antibody relaxed (HAR) and low antibody relaxed (LAR) had been relaxed for 17 generations while HAS and LAS were in generation 40 (Zhao et al., 2012). Chickens were fed antibiotic free corn-soybean mash diets. The starter diet fed to 8 weeks of age contained 20% CP and 2,685 kcal ME/kg of feed. From then to 20 weeks the diet contained 16% CP and 2761 kcal ME/kg. Thereafter, the diet contained 16.1% CP and 2,752 kcal ME/kg. Feed and water were provided *ad libitum*. The chickens were reared in floor pens with wood shavings as liter to 20 weeks of age and transferred to individual cages with wire floors covered with shipping paper to prevent coprophagy. Fresh fecal samples (<1 h) were collected from 114 adult chickens (46 weeks of age) and immediately stored at 4°C with long term storage at −80°C. The groups consisted of 10 HAS males (HASM), 27 HAS females (HASF), 7 LAS males (LASM), 18 LAS females (LASF), 10 HAR males (HARM), 16 HAR females (HARF), 8 LAR males (LARM), and 8 LAR females (LARF).

### Microbiota DNA extraction, 16S rRNA amplification, sequencing, and sequence data processing

Microbiota genome DNA extraction, 16S rRNA amplification and sequencing followed that described in our previous report (Zhao et al., 2013). Microbial genome DNA was extracted from fecal samples using QIAamp DNA stool mini kit (QIAGEN, cat#51504) following the manufacturer's recommendation. Harvested DNA was measured on a NANODROP LITE spectrophotometer to estimate DNA quantity and quality. V4 of 16S rRNA were PCR amplified from microbial genome DNA harvested from fecal samples using barcoded fusion primers (forward primers: 5′ AYTGGGYDTAAAGNG 3′, reverse primers: 5′ TACNVGGGTATCTAATCC 3′) were used for the remainder of our study. The PCR condition and PCR product purification followed our previous publication (Zhao et al., 2013). The purified Barcoded V4 amplicons were sequenced using the pair-end method by Illumina Miseq. Sequencing was carried out by the Shanghai Personal Biotechnology Co., Ltd. The DNA sequences are publicly available in MG-RAST under the project name“high-low-antibody titers.”

Sequences with an average phred score lower than 25, containing ambiguous bases, homopolymer run exceeds 6, having mismatches in primers, or sequence lengths shorter than 100 bp were removed. Only sequences with an overlap longer than 10 bp and without any mismatchs were assembled according to their overlap sequence. Reads which could not be assembled were discarded. Barcode and sequencing primers were trimmed from assembled sequence. Trimmed sequences were uploaded to QIIME for further analysis.

### Taxonomy classification and analysis

The trimmed and assembled sequences from each sample were aligned to the Greengene 16S rRNA training set 10 using the best hit classification option to classify the taxonomy abundance in QIIME (http://qiime.org/index.html) (Caporaso et al., 2010). Bacterial operation taxonomic units (OTU) were generated using the uclust function in QIIME (http://qiime.org/scripts/pick_otus.html). Adonis and ANOSIM were analyzed by QIIME. Ace, chao, simpson and shannon index were calculated by mothur. LEfSe was applied to identify different taxa microbes among lines using the default parameters (LDA Score > 2, *p* < 0.05) (Segata et al., 2011).

### Functional predictions of the gut microbiota

Microbial functions were predicted using PICRUSt (Langille et al., 2013). The OTUs were mapped to gg13.5 database at 97% similarity by QIIME's command “pick_closed_otus.” The OTUs abundance was normalized automatically using 16S rRNA gene copy numbers from known bacterial genomes in Integrated Microbial Genomes (IMG). The predicted genes and their functions were aligned to Kyoto Encyclopedia of Genes and Genomes (KEGG) database and differences among groups were compared through software STAMP (http://kiwi.cs.dal.ca/Software/STAMP) (Parks and Beiko, 2010). Two-side Welch's *t*-test and the Benjamini-Hochberg FDR (*p* < 0.05) correction were used in two-group analysis.

## Results

### Summary of metagenome sequences data

High-throughput sequencing of fecal samples from 114 individual chickens yielded 8927926 reads, with an average of 78,315 sequences reads for each sample (range from 42,077 to 130,028). The average read length was 227 bp with the distributions of sequence lengths showed in Figure S1a. These Operational Taxonomic Units (OTUs) were generated and characterized for different taxonomic levels including domain, phylum, class, order, family, and genus based on Greengene database using QIIME. Taxonomies present in at least ¼ of the total samples were considered as common and their abundance counts were used for further analysis. The OTU numbers were similar for the four lines (Figure S1b). Alpha diversity measured using the Chao, ACE, Simpson and Shannon indices were similar among lines (Figure S1c). A total of 19 phyla, 41 classes, 66 orders, 117 families, and 213 genera were identified in these samples. At the taxa level, consistent with our rank abundance curves (Figure S1d), a majority of microbes were present at low abundance with the greater sequencing coverage. The abundant microbes were identified following the criterion of the abundance beyond 1% of the total DNA sequences.

### Long-term divergent selection for host antibody titers alters gut microbiota community structure

**Figure 1:**
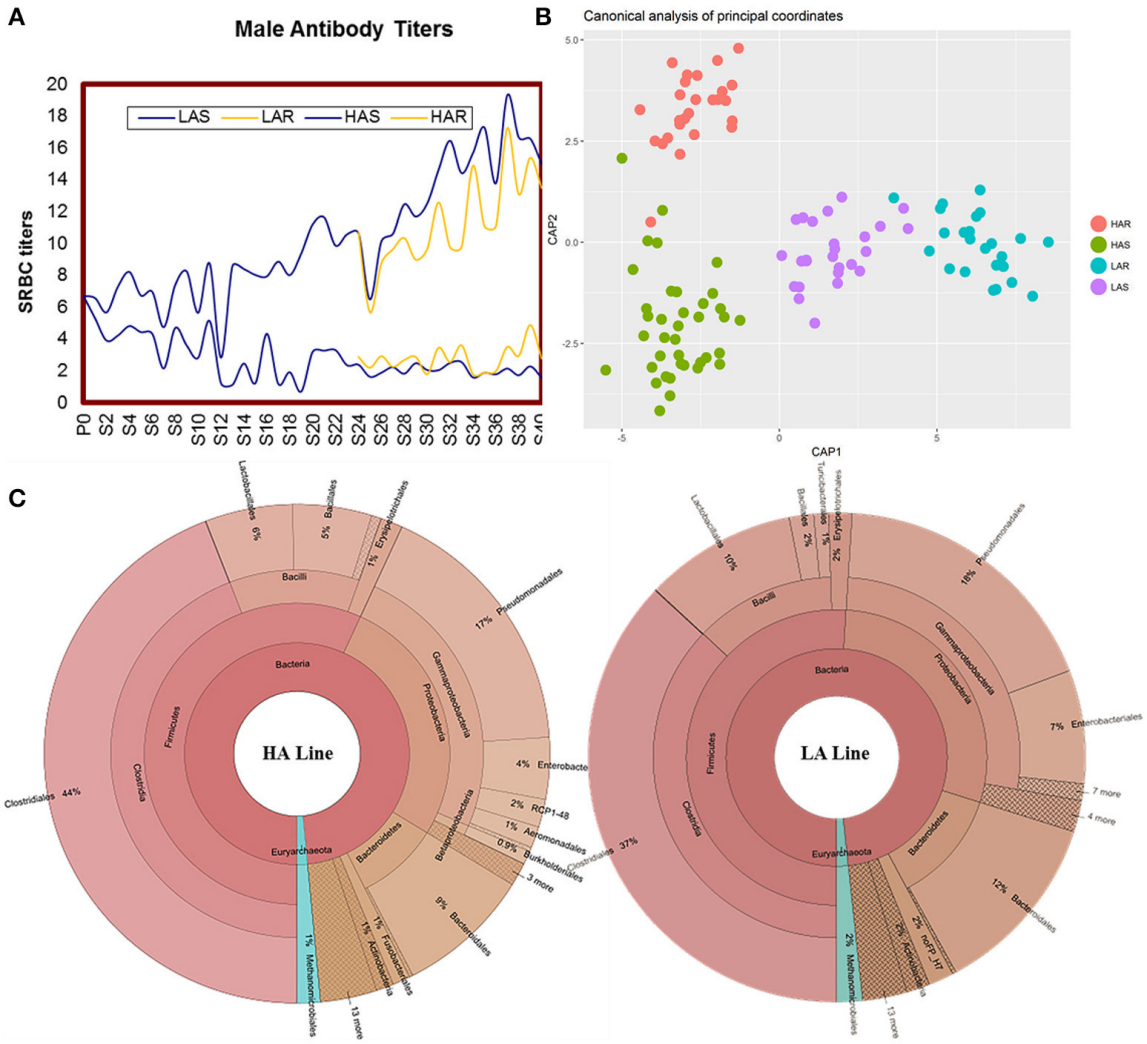
**(A)** Mean sheep red blood cell (SRBC) antibody titers over 40 gene rations for male chickens. Selected lines shown in blue, with relaxed lines shown in yellow. **(B)** Canonical analysis of principal coordinates (CAP) analysis based on unweighted UniFrac metrics. **(C)** The distribution of the gut microbiota composite for HA and LA.

Antibody titers of HAS, LAS, HAR, LAR males are showed across generations in Figure 1A. Comparisons among lines for microbiota were calculated by using UniFrac metrics, which measures phylogenetic dissimilarities among microbial communities (Lozupone and Knight, 2005). Canonical analysis of principal coordinates (CAP) based on the UniFrac metrics revealed a separation among lines (Figure 1B). That antibody titers in the host line were a significant source of variation was evidenced by a nonparametric permutational multivariate analysis, Adonis (*P* = 0.001, HAS vs. LAS, HAR vs. LAR, HAS vs. HAR, LAS vs. LAR). Further confirmation of these results were obtained from ANOSIM (Analysis of similarities) test (*P* = 0.001, *P* = 0.05, *P* = 0.001, and *P* = 0.05).

There was variation of bacterial taxa in the HA and LA lines (Figure 1C). Firmicutes, Proteobacteria and Bacteroidetes, collectively made up more than 90% of the total gut microbiota in each line, with Fusobacteria and Actinobacteria present as minor constituents. For Euryarchaeota, Methanocorpusculum was the dominant phyla that occupied about 1 and 2% in the HA and LA, respectively. At the class level, Clostridia, Gammaproteobacteria, Bacilli and Bacteroidia were the common groups. At the taxonomic order level the major components and respective % for HA and LA were Clostridiales (44 and 37%), Pseudomonadales (17 and 18%), Bacteroidales (9 and 12%), Lactobacillales (6 and 10%), Enterobacteriales (4 and 7%) and Bacillales (5 and 2%). Because both lines originated from the same founder population with the single section criterion being that they were selected for high or low antibody titers to SRBC suggests that the antibody response background of the host has an important role on the population structure of gut microbiota.

### Microbiota which associate with host antibody response

**Figure 2:**
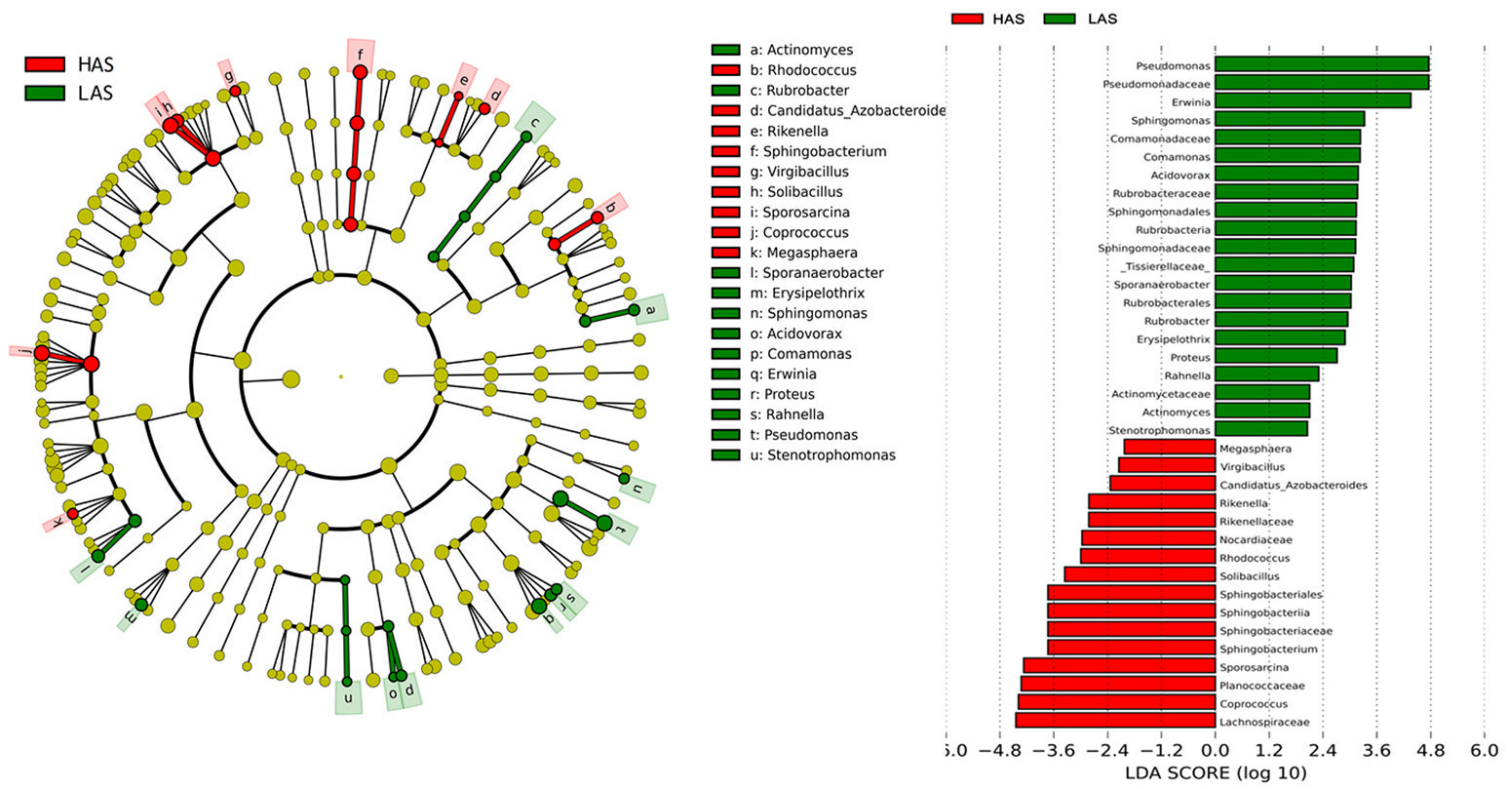
LEfSe identified taxons for HAS and LAS. **(Left)** Taxonomic cladogram. (Red) Taxa enriched in HAS or LAS are colored by red or green, respectively. **(Right)** HAS-enriched taxa are shown with a positive LDA score (green), and taxa enriched in LAS are shown with a negative score (red). Only taxa meeting an LDA significant threshold > 2 and *p* < 0.05 are shown.

**Figure 3:**
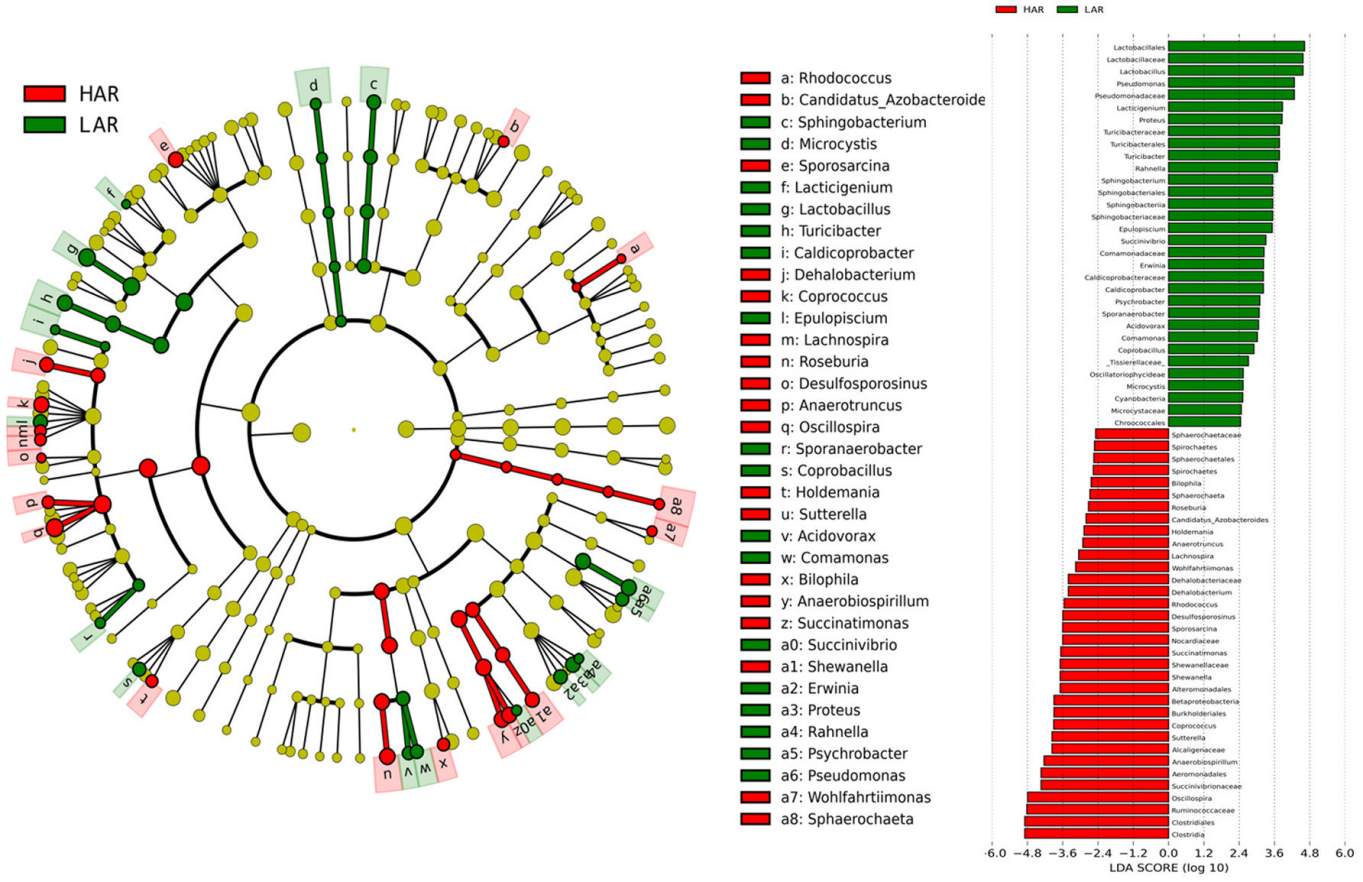
LEfSe identified taxons forHAR and LAR. **(Left)** Taxonomic cladogram. Taxa enriched in HAR or LAR are colored by red or green, respectively. **(Right)** HAR-enriched taxa are shown with a positive LDA score (green), and taxa enriched in LAR are shown with a negative score (red). Only taxa meeting an LDA significant threshold > 2 and *p* < 0.05 are shown.

To identify specific bacterial taxa that are associated with background antibody response, we compared the fecal microbiota in HA (HAS and HAR) and LA (LAS and LAR) using LEfSe. A cladogram representative of the structure of the host-microbiota axis (Figures 2, 3) showed a significant shift of microbiota between HA and LA. At the phylum level, two and four phylum were greater in LAS and HAR relative to the HAS and LAR (Table S1). At the level of order, compared to the HA (HAR and HAS) (Table S2), the abundance of Aeromonadales, and Burkholderiales were lower and Lactobacillales was higher in LAR (Figure S2a). Only Enterobacteriales increased in LAS. At the family level (Table S3), the abundant family, Alcaligenaceae, Ruminococcaceae and Succinivibrionaceae were prevalent in HAR, while Lactobacillaceae, and Pseudomonadaceae were enriched in LAR. Enterobacteriaceae, BS11 and Pseudomonadaceae were enriched in LAS while conversely Planococcaceae was increased in HAS (Figure S2b).

**Figure 4:**
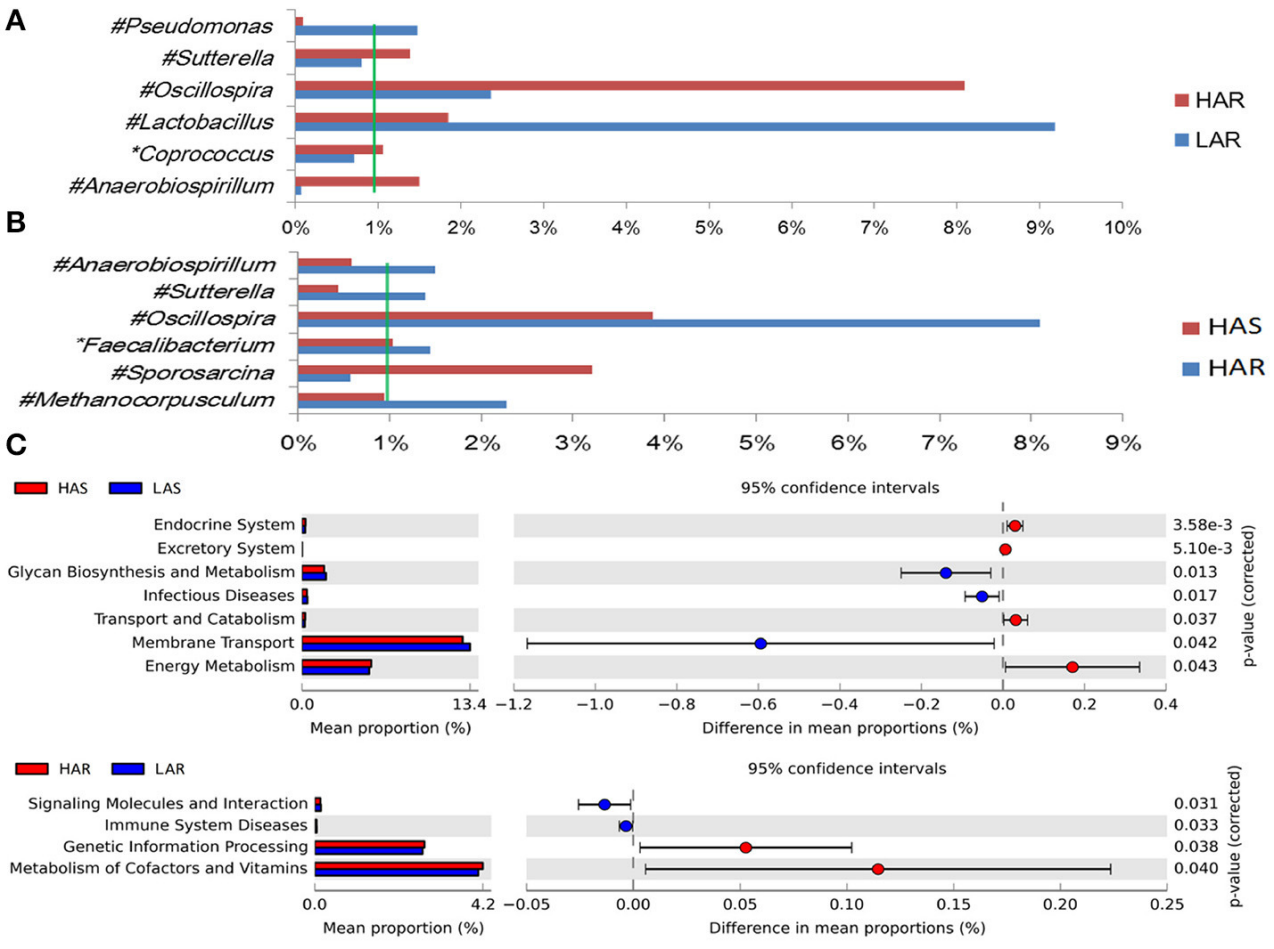
Comparisons of relative abundance at the abundant bacterial genus levels between: **(A)** HAR vs. LAR, **(B)** HAR and LAR; ^*^p < 0.05; #*p* < 0.01. **(C)** Comparisons of functional pathway between microbes of HAS vs. LAS and HAR vs. LAR.

The number of genera that differed (*P* < 0.05) for HAR vs. LAR and HAS vs. LAS were 35 and 21, respectively (Table S4 and Table 1). Among the abundant genera, four (*Anaerobiospirillum, Coprococcus, Oscillospira* and *Sutterella*) increased in HAR and two (*Lactobacillus* and *Pseudomonas*) were enriched in LAR (Figure 4A). *Pseudomonas* and *Erwinia* decreased in HAS, while *Sporosarcina* and *Coprococcus* decreased in LAS (Figure S2c). Among genera, all 8 belonging to Proteobacteria were enriched in LAS (Table 1), while for the relaxed lines HAR and LAR the respective numbers were 6 and 8 (Table S4). In addition, as shown in Figure 4A and Table S4, butyrate-producing bacteria *Oscillospira* increased dramatically (LDA Score = 4.80, 2.3% in LAR and 8.1% in HAR) in HAR, while the abundance of *Lactobacillus* increased in LAR (LDA Score = 4.57, 1.8% in HAR and 9.1% in LAR).

**Table 1:** Relative abundance of microbiota at the genus level for HAS (high antibody selected) and LAS (low antibody selected) lines.

**Phylum**	**Genus**	**Line^a^**	**LDA Score**	***p*-value**
Actinobacteria	*Actinomyces*	LAS	2.102	0.042^*^
	*Rhodococcus*	HAS	3.001	0.044^*^
	*Rubrobacter*	LAS	2.954	0.040^*^
Bacteroidetes	*Candidatus_Azobacteroides*	HAS	2.343	0.019^*^
	*Rikenella*	HAS	2.821	0.039^*^
	*Sphingobacterium*	HAS	3.732	0.025^*^
Firmicutes	*Coprococcus*	HAS	4.390	0.001^**^
	*Erysipelothrix*	LAS	2.893	0.036^*^
	*Megasphaera*	HAS	2.028	0.008^**^
	*Solibacillus*	HAS	3.358	0.024^*^
	*Sporanaerobacter*	LAS	3.029	0.040^*^
	*Sporosarcina*	HAS	4.271	0.025^*^
	*Virgibacillus*	HAS	2.151	0.007^**^
Proteobacteria	*Acidovorax*	LAS	3.183	0.001^**^
	*Comamonas*	LAS	3.226	0.001^**^
	*Erwinia*	LAS	4.357	0.003^**^
	*Proteus*	LAS	2.712	0.000^**^
	*Pseudomonas*	LAS	4.756	0.001^**^
	*Rahnella*	LAS	2.304	0.002^**^
	*Sphingomonas*	LAS	3.324	0.027^*^
	*Stenotrophomonas*	LAS	2.050	0.010^*^

* *p < 0.05*,

** *p < 0.01*.

a *The line with the significantly greater abundance*.

### Dynamic distribution of gut microbiota when selection relaxed

**Figure 5:**
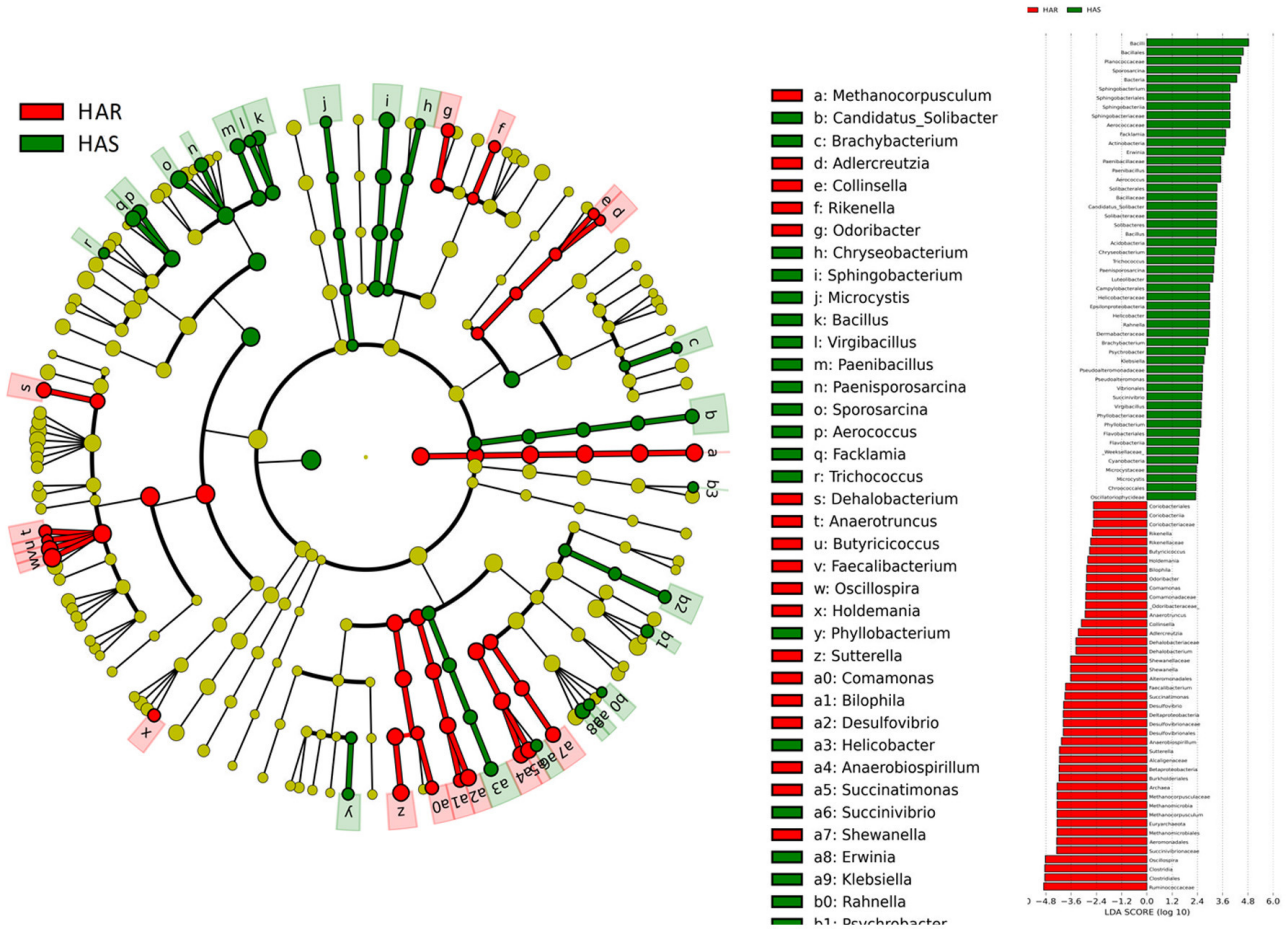
LEfSe identified taxons for HAS and HAR. **(Left)** Taxonomic cladogram. Taxa enriched in HAR or HAS are colored by red or green, respectively. **(Right)** HAS-enriched taxa are shown with a positive LDA score (green), and taxa enriched in HAR are shown with a negative score (red). Only taxa meeting an LDA significant threshold > 2 and *p* < 0.05 are shown.

**Figure 6:**
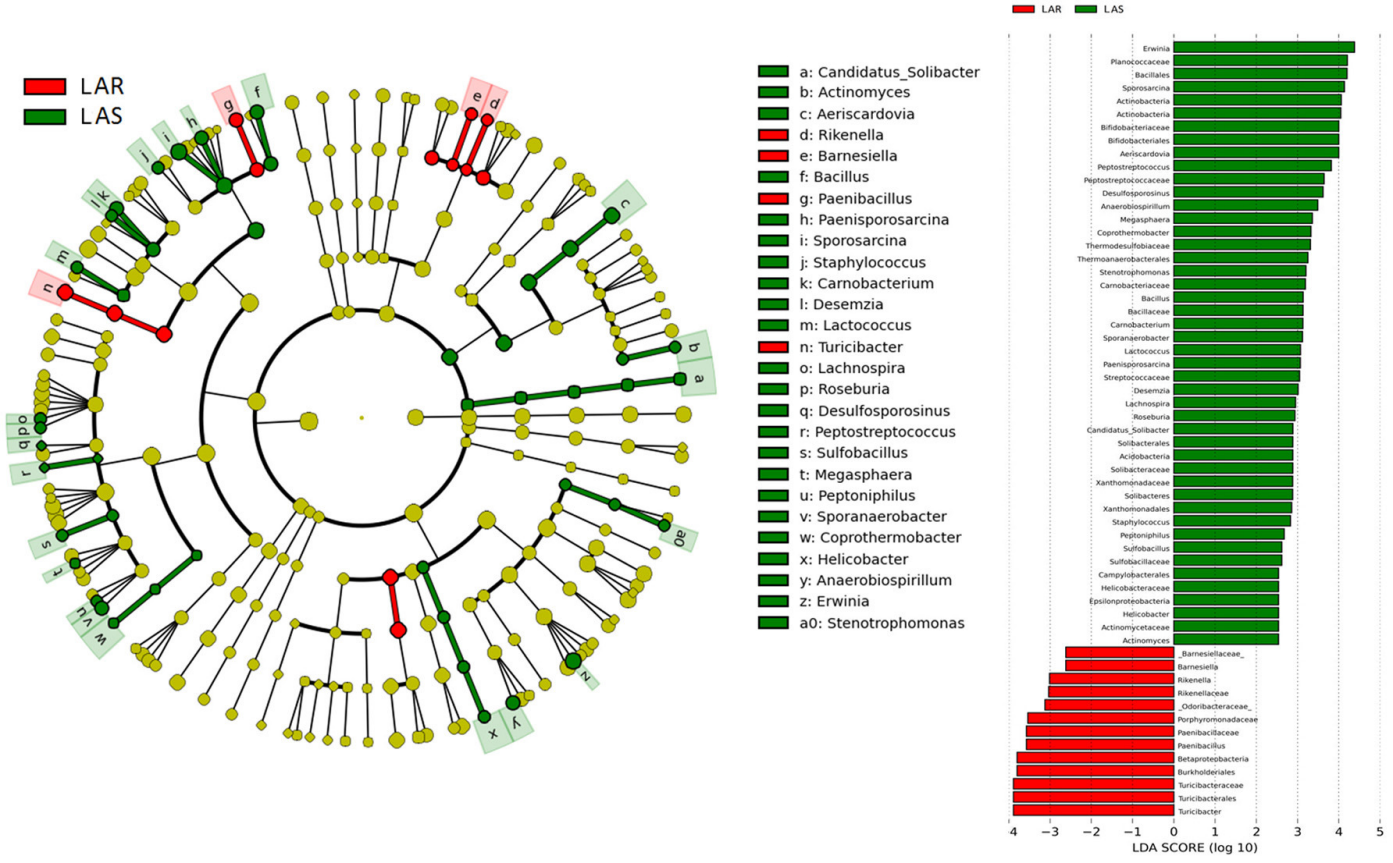
LEfSe identified taxons for LAS and LAR. **(Left)** Taxonomic cladogram. Taxa enriched in LAR or LAS are colored by red or green, respectively. **(Right)** LAS-enriched taxa are shown with a positive LDA score (green), and taxa enriched in LAR are shown with a negative score (red). Only taxa meeting an LDA significant threshold > 2 and *p* < 0.05 are shown.

The greatest differences between the selected and their respective relaxed lines in taxa with key markers at different phylogenetic levels are seen in Figures 5, 6. While eight phyla were significantly different between HAS and HAR, only one was different between LAS and LAR (Table S5). There were seven abundant orders differed between HAS and HAR (Table S6). Specifically, Bacillales, Lactobacillales and Pseudomonadales were enriched in HAS (Figure S3a). While only Bifidobacteriales and Bacillales increased in LAS and Burkholderiales was enriched in LAR (Figure S3a). At the family level (Table S7), levels of Ruminococcaceae (LDA Score = 4.51, 18.9% in HAR and 8.6% in HAS) and Succinivibrionaceae increased in HAR and Moraxellaceae and Planococcaceae was enriched in HAS (Figure S3b). In contrast, only Bifidobacteriaceae was higher in LAS than LAR.

Thirty-nine genera were significantly different between HAS and HAR (Table S8). Among them, 5 abundant genera (*Oscillospira, Methanocorpusculum, Anaerobiospirillum, Faecalibacterium*, and *Sutterella*) were enriched in HAR and *Sporosarcina* increased in HAS (Figure 4B). Except for *Faecalibacterium* and *Oscillospira*, two relatively lower abundance genera (*Anaerotruncus* and *Butyricicoccus*) that belonged to Ruminococcaceae were also enriched in HAR (Table S8). Ruminococcaceae is a butyrate-producing bacterium containing four genera that increased considerably in HAR. The abundant genera were *Faecalibacterium* (LDA Score = 3.87, 1.4% in HAR and 1.0% in HAS) and *Oscillospira* (LDA Score = 4.83, 8.1% in HAR and 3.9% in HAS) (Figure 4B).

Twenty-seven genera were significantly different between LAS vs. LAR. Among them, the abundant genera (*Aeriscardovia, Erwinia*, and *Sporosarcina*) increased in LAS, and only *Turicibacter* enriched in LAR (Figure S3c). Excepting *Paenibacillus, Turicibacter, Barnesiella*, and *Rikenella*, the remainder of the 23 genera significantly decreased in LAR (Table S8).

### Sex influences composition of gut microbiota

There were significant differences between sexes at several levels of taxa (Figure S4). Among the 4 lines, there was sexual dimorphism for 12, 11, 10, and 11 genera (Table S9). *Succinatimonas* (HARM vs. HARF) and *Turicibacter* (LARM vs. LARF) were the abundant genus and influenced by sex. Microbial functions involved pathways relating to Glycosphingolipid biosynthesis—lacto and neolacto series, Cyanoamino acid metabolism, Arginine and proline metabolism, Glutamatergic synapse and Betalain biosynthesis were enriched more in females than males. While the abundance of steroid biosynthesis, ubiquinone and other terpenoid-quinone biosynthesis, bacterial invasion of epithelial cells, caffeine metabolism, lipoic acid metabolism and prion diseases were higher in males than females (Figure S5a).

## Discussion

**Figure 7:**
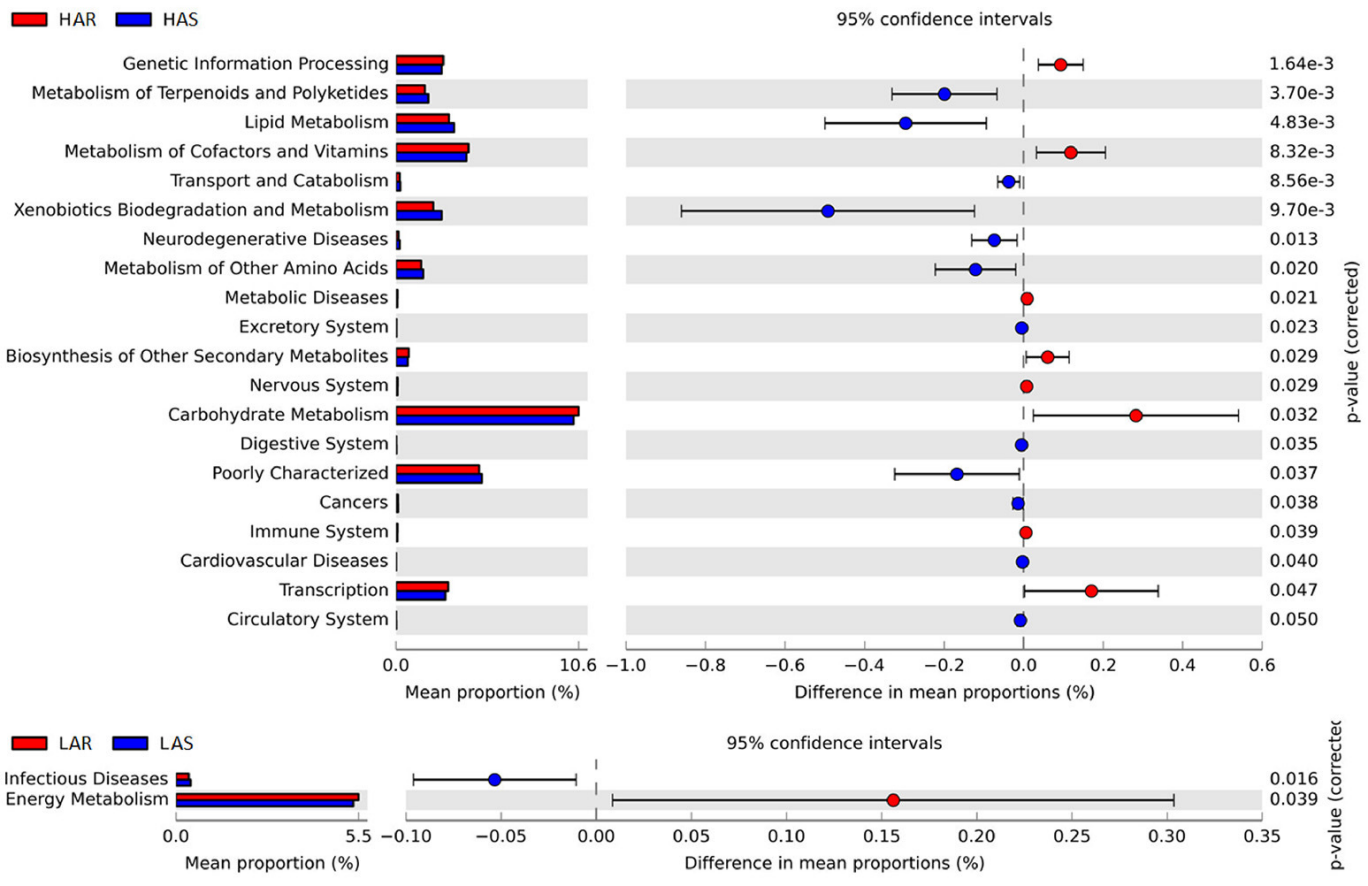
Comparisons of functional pathways between microbes of HAS vs. HAR **(Top)** and LAS vs. LAR **(Bottom)**. The pathways shown in the figure were predicted by PICRUSt.

The microbiota can induce adaptive changes in host immunity (Farkas et al., 2015), and our results show that the lines selected for differences in immune response contribute to adaptive changes in the microbiota. To further investigate the association of microbiota functions for high vs. low and selected vs. relaxed lines, we used PICRUSt to produce predicted metagenomes from 16S rRNA gene sequence data and then applied STAMP to estimate differences in predicted abundances of KEGG. From this analysis we observed differences between the lines in the endocrine system, excretory system, transport and catabolism, energy metabolism, genetic information processing, and, metabolism of cofactors and vitamins were differed (HA > LA). Pathways allocated for signaling molecules and interactions (LAR), immune system diseases (LAR), glycan biosynthesis and metabolism (LAS), infectious diseases (LAS) and membrane transport (LAS) were different between the lines (HA < LA) (Figure 4C). Furthermore, the three pathways of primary bile acid biosynthesis, secondary bile acid biosynthesis and systemic lupus erythe matosus related to immune system were enriched in LAR (Figure S5b). For selected vs. relaxed lines, microbiota in the former had a greater abundance of functions involved in metabolic pathways involving infectious diseases (LAS line), metabolism of Terpenoid and polyketides, lipid metabolism, transport and catabolism, xenobiotic biodegradation and metabolism, neurodegenerative diseases, metabolism of other amino acids, excretory system, digestive system, cancers, cardiovascular diseases and circulatory system than the later. In contrast, the relaxed lines contained higher energy metabolism, genetic information processing, metabolism of cofactors and vitamins, metabolic diseases, biosynthesis of other second metabolites, nervous system, carbohydrate metabolism, immune system (HAR line) and transcription than their respective selected lines (Figure 7). Taken together, the analysis of microbial function revealed that the pathway of infectious diseases enriched in LAS (Figure 4C). When selection was relaxed, enriched pathway of infectious diseases was replaced by immune system diseases in LAR (Figure 4C).

In this study, the high and low selected and relaxed lines of chickens that originated from a common founder population of chickens (Siegel and Gross, 1980; Siegel et al., 1982; Boa-Amponsem et al., 1997; Dorshorst et al., 2011; Zhao et al., 2012; Farkas et al., 2015; Lillie et al., 2017) provide evidence for humoral immunity-microbe co-microevolution. Dramatic differences in gut microbiota were observed in these lines that had undergone long-term artificial and relaxed selection for high or low antibody titers to SRBC (Figure 1B). Twenty-one genera were identified as significantly different between the high and low selected lines with 8 genera that belonged to Proteobacteria all enriched in the LAS (Table 1). The Proteobacteria is a major phylum of Gram-negative bacteria. They include a wide variety of pathogens and have an outer membrane composed mainly of lipopolysaccharides (Matsuura, 2013) which acts as aprototypical endotoxin caused immunity responses (Conde-Álvarez et al., 2012; O'Neill et al., 2013). The phenomenon is consistent with other studies, such as, human immunodeficiency virus (HIV) infection which is a chronic illness characterized by progressive CD4^+^ T cell loss and associated with increases in Proteobacteria (potentially pathogenic taxa) in the gut microbiota (Dinh et al., 2014; Mutlu et al., 2014; Serrano-Villar et al., 2016). In addition, microbial functions also revealed that pathways of infectious diseases were enriched in LAS, results consistent with those observed at the taxa level. The long-term selection for antibody response to SRBC, resulted in lines that exhibited antibody responses which may inhibit colonization of certain types of bacteria. Then when selection was relaxed, there was considerable shifting of gut microbiota where 35, 39, and 27 genera were significantly different in comparisons of HAR vs. LAR, HAS vs. HAR, and LAS vs. LAR. Concomitantly, variation in antibody titers was also observed, inferring that in the process of co-microevolution, under the artificial selection, variation of antibody titers dominated the microorganisms to colonize in host gut. Conversely, the shift in genera that occurred when selection was relaxed may contribute to reconstructed antibody responses.

When selection was relaxed, there was opportunity for adaptive changes according to the pressure of natural selection (Figure 1A). Thus, the fragile immune homeostasis constructed by artificial selection had costs with natural selection for an intermediate optimum for immune homeostasis. There is evidence that the gut microbiota can induce adaptive change of host immunity. For example, flagellin stimulated intestinal CD103^+^ CD11b^+^ dendritic cells produce Interleukin 23 (IL-23) which enhance mucosal innate immune defenses (Kinnebrew et al., 2012) and regulate TLR4 gene expression by modifications of the level of methylation in 5′ region of the promotor by commensals (Takahashi et al., 2011). In our study, the butyrate-producing bacteria *Oscillospira* and Ruminococcaceae (*Faecalibacterium, Oscillospira, Anaerotruncus*, and *Butyricicoccus*) were greater in HAR than LAR and HAS (Figures 4A,B, Figure S3b and Table S8), respectively. Butyrate, a main type of short-chain fatty acids (SCFA), has been involved in the regulation of macrophage function by inhibiting histone deacetylases (Chang et al., 2014). In particular, butyrate endows dendritic cells with a superior ability to facilitate regulatory T cells differentiation (Wang et al., 2009; Arpaia et al., 2013), or inhibits histone deacetylase influence regulatory T cells by binding to GPCR43 (Tao et al., 2007; Smith et al., 2013). In some autoimmune diseases with aberrant antibody production, there is a decrease in the function or number of regulatory T cells (de Lafaille and Lafaille, 2002; Balandina et al., 2005; Mqadmi et al., 2005).

Regulatory T cells have a critical role in immune homeostasis and in suppressing excessive immune responses by the host (Sakaguchi et al., 2008). For example, regulatory T cells can inhibit IgG production of systemic lupus erythe matosus (SLE), a form of an antibody-driven autoimmune disorder (Facciotti et al., 2016). In addition, regulatory T cells can indirectly suppress Ig production via regulating T helpers cell in stimulating B cells (Lim et al., 2004), or by directly restraining Ig production dependent on B cell responses accompanied by inhibition of Ig class switch recombination (Lim et al., 2005). The cell wall components of *Lactobacillus* contain microorganism-associated molecular patterns (MAMPs) that are recognized by specific pattern recognition receptors (PRRs) that are expressed in the intestinal mucosa of the host (Wells et al., 2011). They include Toll-like receptors (TLRs), NOD-like receptors (NLRs) and C-type lectin receptors (CLRs). Through these signal pathways *Lactobacillus* is involved in the regulation of the immune system of host. For example, *Lactobacillus* species may deliver signals in dendritic cells (DCs) through TLR-2, thereby promoting the activation of CD4^+^ and CD8^+^ T cells to T-helper cells (Cella et al., 1997; Mohamadzadeh et al., 2005). Depending on the pattern, DCs activate and expand T-helper cells, which in turn induce B-cell growth and antibody production (Dubois et al., 1997; Banchereau and Steinman, 1998). In addition, some *Lactobacillus* species can increase Ig production (Rizzardini et al., 2012; Nishihira et al., 2016).

Microbiota functions associated with primary bile acid biosynthesis, secondary bile acid biosynthesis and systemic lupus erythe matosus were relatively greater in LAR than the other lines (Figure S5b). Bile acids can be sensed by a subset of nuclear receptors including the farnesoid X receptor (FXR) (Vavassori et al., 2009) and G-protein coupled receptor 5 (TGR 5) (Keitel et al., 2007) to modulate the immunity system. Moreover, systemic lupus erythe matosus is associated by an excessively high production of Ig (Facciotti et al., 2016). Implying that the microbes in LAR may contribute to greater production of Ig and thus the gut microbiota, especially *Oscillospira* and *Lactobacillus* may play a key role in immune homeostasis. Through bidirectional selection from a common founder population for humoral immunity, changes occurred in the host microbiota axis which in turn became more balanced when selection was relaxed. The bidirectional interplay demonstrated changes in the microbiota as correlated responses. When selection was relaxed the microbiota coevolved reflecting a coevolution of host and gut microbiota. Such a dynamic linkage between humoral immunity and gut microbial ecology, together with our results, indicates that manipulation of gut microbial communities could be another approach in the treatment of autoimmune diseases.

## Author contributions

LY, PS, YZ, and HM performed the experiments. SL, JD, RD, CFH, CH, and KX contributed to some of the experiments. LY, PS, YZ, and HM designed experiments, analyzed data, and wrote the paper.

### Conflict of interest statement

The authors declare that the research was conducted in the absence of any commercial or financial relationships that could be construed as a potential conflict of interest.
