# Supplementary material for: Gut Microbiota Co-microevolution with Selection for Host Humoral Immunity
Source: Front Microbiol. 2017 Jul 4;8:1243. doi: 10.3389/fmicb.2017.01243 (PMC5495859; doi:10.3389/fmicb.2017.01243)

Figure S1: (a) The length distribution of sequence reads. (b) Venn diagram of OTUs of fecal microbiota in the four lines. (c) Alpha diversity of HA and LA. (d) Rarefaction curves (at a 97% similarity level) of fecal microbiota.

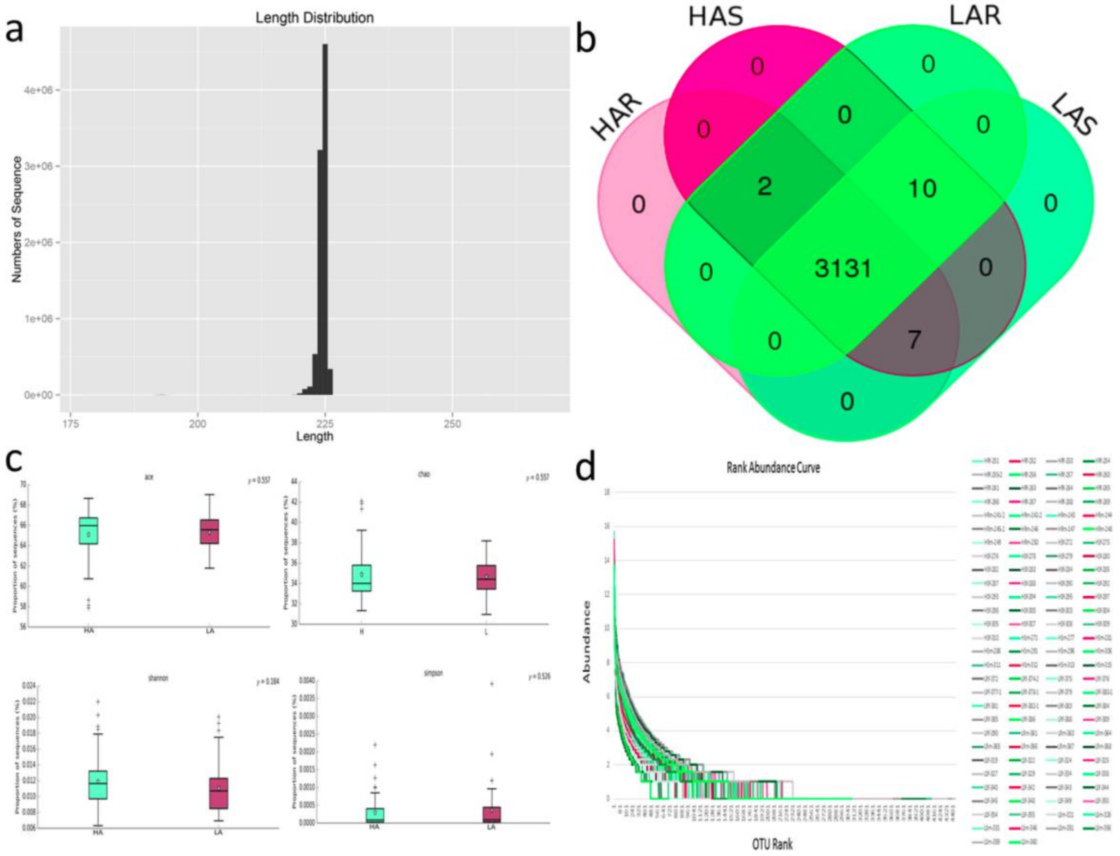

Supplement: Supplementary file 10 [file Image1.PDF]
