# Supplementary material for: Gut Microbiota Co-microevolution with Selection for Host Humoral Immunity
Source: Front Microbiol. 2017 Jul 4;8:1243. doi: 10.3389/fmicb.2017.01243 (PMC5495859; doi:10.3389/fmicb.2017.01243)

Figure S2: The differently abundant bacterial taxa for HAS vs LAS and HAR vs LAR. (a) At the order level. (b) At the family level. (c) At the genus level.*: $p < 0.05$; #: $p < 0.01$.

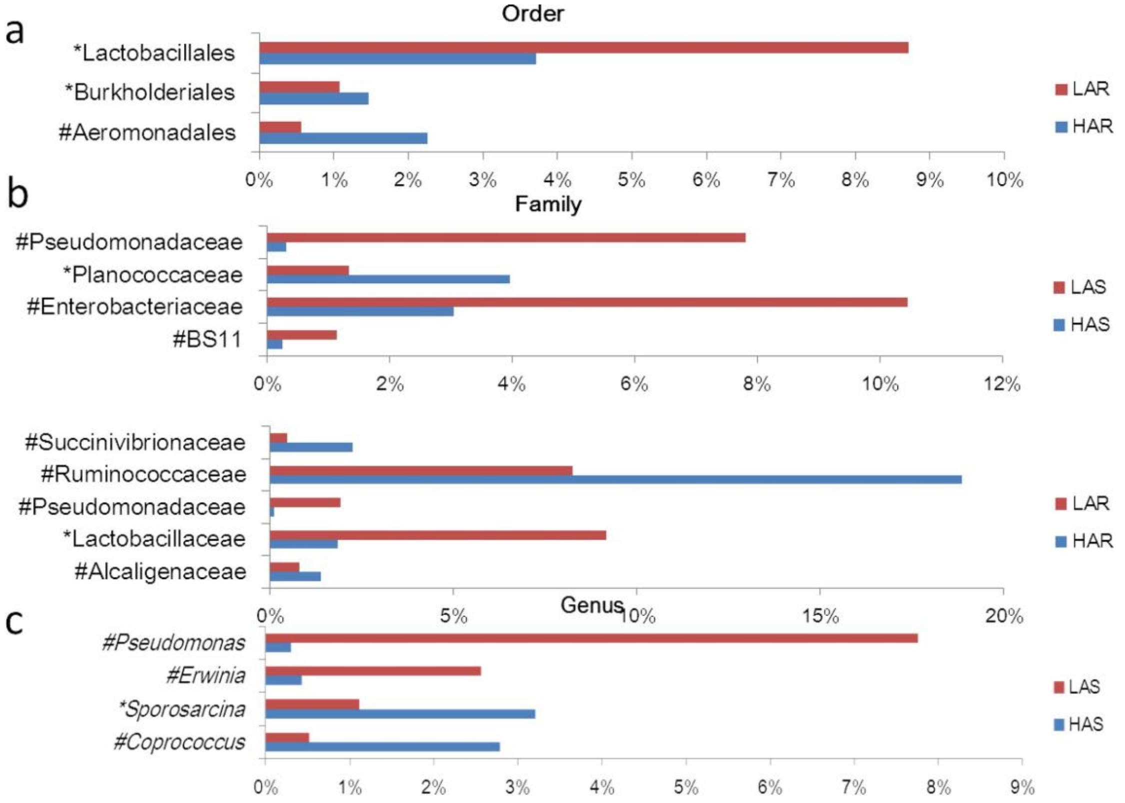

Supplement: Supplementary file 11 [file Image2.PDF]
