# Supplementary material for: Gut Microbiota Co-microevolution with Selection for Host Humoral Immunity
Source: Front Microbiol. 2017 Jul 4;8:1243. doi: 10.3389/fmicb.2017.01243 (PMC5495859; doi:10.3389/fmicb.2017.01243)

Figure S5: Comparisons of functional pathways between: (a) Microbes of females and males. (b) For microbes of HAR and LAR at level 3 of KEGG pathway analysis

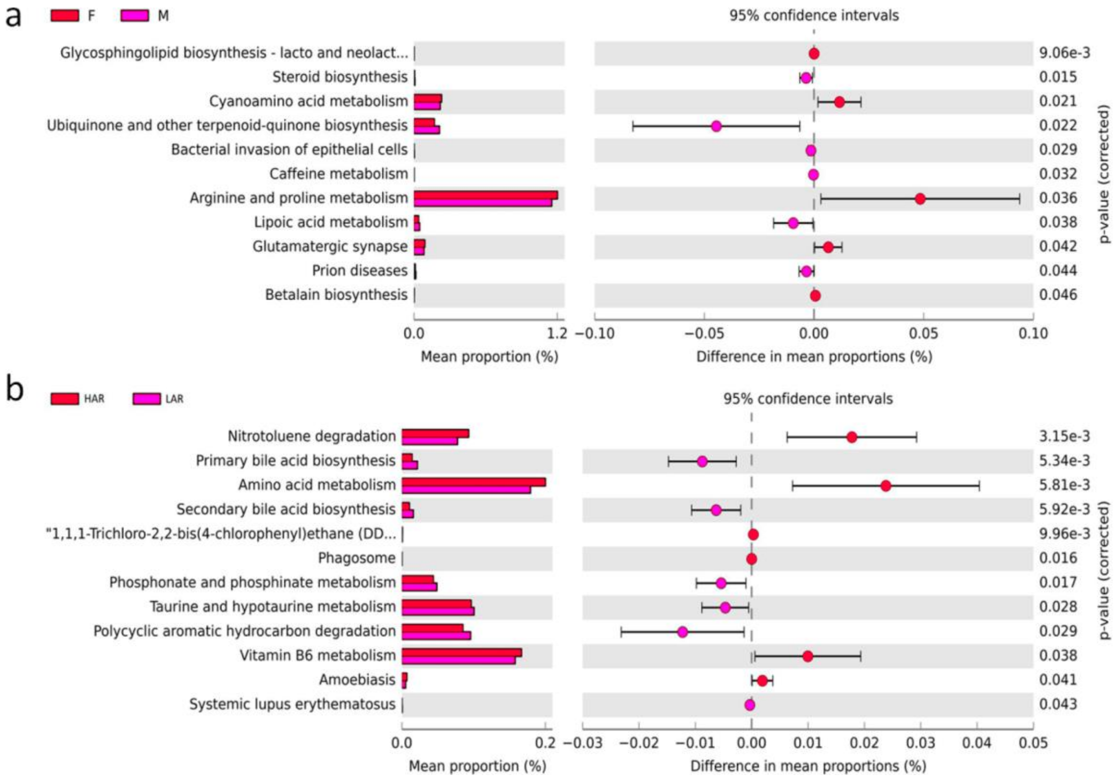

Supplement: Supplementary file 14 [file Image5.PDF]
