# Supplementary material for: Gut Microbiota Co-microevolution with Selection for Host Humoral Immunity
Source: Front Microbiol. 2017 Jul 4;8:1243. doi: 10.3389/fmicb.2017.01243 (PMC5495859; doi:10.3389/fmicb.2017.01243)

Figure S4: Taxonomic cladogram of LEfSe that identified the most different taxons for females and males. Taxa enriched for females or males were colored by red or green, respectively.

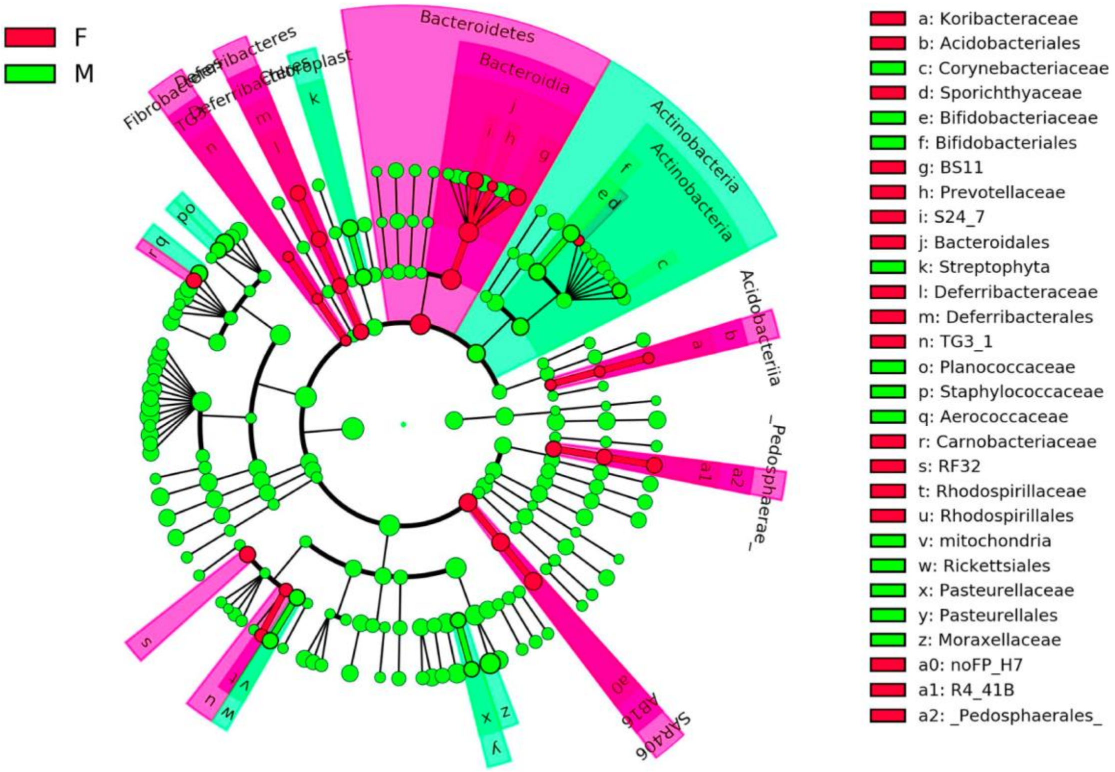

Supplement: Supplementary file 13 [file Image4.PDF]
